# Anti-mullerian hormone is expressed by endometriosis tissues and induces cell cycle arrest and apoptosis in endometriosis cells

**DOI:** 10.1186/1756-9966-33-46

**Published:** 2014-05-29

**Authors:** Pietro G Signorile, Francesca Petraglia, Alfonso Baldi

**Affiliations:** 1 Centro Italiano Endometriosi, Via Aurelia, 559, Rome 00165, Italy

**Keywords:** Endometriosis, Immunohistochemistry, AMH, Apoptosis

## Abstract

**Background:**

The anti-mullerian hormone (AMH) is a member of the transforming growth factor β (TGF-β) superfamily, which is responsible of the regression of the mullerian duct. AMH is expressed in the normal endometrium, where, acting in a paracrine fashion, negatively regulates cellular viability. Our objective was to evaluate the in vitro effects of the treatment with AMH of endometriosic cells.

**Methods:**

AMH expression in human endometriosis glands was evaluated by immunohistochemistry. RT-PCR has been used to quantify the expression levels of AMH and AMH RII isoforms, as well as of cytochrome P450 in both endometriosis epithelial and stromal cells Effects of AMH and AMH-cleaved treatment in endometriosis cells were evaluated by flow-cytometry analysis. Finally, it has been evaluated the effect of plasmin-digested AMH on cytochrome P450 activity.

**Results:**

AMH and AMH RII isoforms, as well as cytochrome P450, were expressed in both endometriosis epithelial and stromal cells. Treatment of endometriosis stromal and epithelial cell growth with AMH was able to induce a decrease in the percentage of cells in S phase and increase percentage of cells in G1 and G2 phase; coherently, decreased cell viability and increased percentage of cells death fraction was observed. The plasmin-digested AMH was able to suppress most of the cytochrome P450 activity, causing an increase of pre-G1 phase and of apoptosis induction treating with plasmin-digested AMH in both cell lines, most marked in the epithelial cells.

**Conclusions:**

The data produced suggest a possible use of AMH as therapeutic agents in endometriosis.

## Background

Endometriosis is a gynaecological disease defined by the histological presence of endometrial glands and stroma outside the uterine cavity. Most commonly, endometrial structures are implanted over visceral and peritoneal surfaces, but rarely also in the pericardium, pleura, and even brain [1]. The prevalence in the general female population is 6-10%; in women with pain, infertility or both, the frequency increases to 35-60% [2]. Endometriosis is usually associated with infertility and pelvic pain such as chronic dysmenorrhea, intermestrual abdominal and pelvic pain, back pain, dysuria, dyschezia and dyspareunia [3]. Moreover, it is often associated with a decrease of ovarian reserve and reduction of ovarian volume [4]. Despite the fact that this disease is quite common among women, it is frequently misdiagnosed, the pathogenesis is unknown and the diagnostic and therapeutic protocols are still not fully adequate [1,3]. Currently, none of the pathogenetic theories proposed, such as retrograde menstruation, coelomic metaplasia or staminal cells, has definitively been proved [1]. Interestingly, our research group has recently demonstrated the presence of endometrial implants outside the uterus in a significant number of female human fetuses, thus demonstrating that alterations in the fine-tuning of the primitive mullerian tube formation is one of the causes of endometriosis [5-9].

The anti-mullerian hormone (AMH) is a homodimeric glycoprotein member of the transforming growth factor β (TGF-β) superfamily, which is secreted by Sertoli cells in the embryonic testes and is responsible of the regression of the mullerian duct [10]. In the female fetus ovarian granulosa cells begin to secrete low levels of AMH starting from the 32 week of gestation. Levels surge at the time of puberty to approximately 5-8 ng/mL but then gradually decline throughout reproductive life until they become undetectable by menopause. Therefore, AMH levels are considered good indicators of the ovarian reservoir [11]. Recent studies have demonstrated that AMH, as well as AMHRII (one of its receptors), are expressed in the adult female also in the endometrium, where, probably, act in a paracrine fashion and that negatively regulates cellular viability in the endometrium [12].

Leaving from this background, we decided to deeply investigate the potential role of AMH in regulating cell viability and proliferation of endometriosis cells, taking advantage of an in vitro model of epithelial and stromal endometriosis cells, recently generated in our laboratory [13].

## Methods

### Patients and tissue samples

Retrospective evaluation was performed on surgical specimens from 30 patients who underwent surgery for infertility, pelvic pain symptoms (including dysmenorrhea, deep dyspareunia and no-menstrual pain) or adnexal masses between 2009 and 2012 at the “Centro Italiano Endometriosi” in Rome and that were diagnosed with deep infiltrating endometriosis. Representative sections of each specimen were stained with haematoxylin-eosin to confirm the diagnosis of endometriosis. For immunohistochemistry 5-7 μm specimen sections embedded in paraffin, were cut, mounted on glass and dried overnight at 37°C. All sections were then deparaffinized in xylene, rehydrated through a graded alcohol series and washed in phosphate-buffered saline (PBS). PBS was used for all subsequent washes and for antiserum dilution. Tissue sections were quenched sequentially in 3% hydrogen peroxide in aqueous solution and blocked with PBS-6% non-fat dry milk (Biorad, Hercules, CA, U.S.A.) for 1 h at room temperature. Slides were then incubated at 4°C overnight at 1:100 dilution with a rabbit polyclonal antibody for AMH (Abcam, Cambridge, UK). After three washes in PBS to remove the excess of antiserum, the slides were incubated with diluted goat anti-rabbit biotinylated antibody (Vector Laboratories, Burlingame, CA, U.S.A.) at 1:200 dilution in PBS-3% non-fat dry milk (Biorad) for 1 h. All the slides were then processed by the ABC method (Vector Laboratories) for 30 min at room temperature. Diaminobenzidine (Vector Laboratories) was used as the final chromogen and hematoxylin was used as the nuclear counterstain. Negative controls for each tissue section were prepared by leaving out the primary antiserum. All samples were processed under the same conditions. Experiments were performed in compliance with the Helsinki Declaration and the protocols were approved by the ethics committee of the Fondazione Italiana Endometriosi.

### Cell lines and primary cells

Human endometriosis stromal and epithelial cells were described elsewhere [13]. Cells were grown following standard procedures and were propagated in DMEM/F12 (1:1) with 10% Fetal Bovine Serum (FBS) (Gibco, Life Technologies Italia, Monza, Italy), 2 mM L-Glutamine (Euroclone S.p.a, Piero, Italy) and antibiotics (100 U/mL penicillin, 100 μg/mL streptomycin and 250 ng/mL amphotericin-B).

### In vitro treatment with AMH

Cultured human endometrial stromal and epithelial cells were treated with Recombinant Human Mullerian-Inhibiting Substance (rhMIS)/anti-Mullerian hormone (AMH) - E-Coli derived (R&D Systems) and Purified recombinant protein of Homo sapiens AMH (OriGene Technologies, Rockville, MD, USA) at three different final concentrations (10-100-1000 ng) for three different time (24-48-72 hrs). Plasmin-cleaved AMH was used instead of the full-length molecule for incubation times indicated. AMH was digested by Plasmin from human plasma (Sigma-Aldrich, Italia) 1 h at 37°C in a ratio of 25 to 1, as described [14]. The effect of AMH on the activity of cytochrome P450 aromatase (CYP19) was measured through the P450-Glo assays (Promega Italia, Milano, Italy) [15].

### Cell vitality

Experiments were performed in duplicate. Cells were diluted 1:1 in Trypan blue (Sigma-Aldrich, Italia) and counted.

### Cell cycle and cell death

Analysis was performed in duplicate. 100.000 cells were re-suspended in the staining solution containing RNAse A, Propidium Iodide (PI) (50 mg/mL), sodium citrate (0.1%), and NP40 (0.1%) in PBS 1X for 30 min in the dark and room temperature. Cell cycle distribution was assessed with an FACScalibur flow cytometer (Becton Dickinson), and 10,000 cells were analyzed by ModFit version 3 Technology (Verity) and Cell Quest (Becton Dickinson) [16].

### RNA, RT-PCR

Total RNA was extracted with TRIzol (Life Technologies) and converted into cDNA using SuperScript VILO kit according to the manufacturer’s protocol. (Invitrogen). Converted cDNA was amplified using EuroTaq (Euroclone). Amplified DNA fragments were loaded on 2.0% agarose gel and photographed on a Gel Logic 200 Imaging system UV transilluminator (Kodak). Levels of AMH, AMH type II Receptor (AMHR-II) and CYP19 expression were quantified by Reverse Transcription Polymerase Chain Reaction (RT-PCR). Real-Time PCR was performed using iQ_ SYBR_ Green Supermix (Bio-Rad) in a DNA Engine Opticon2 thermal cycler (MJ Research Incorporated). Primers: AMH gene (1) (Forward 5′-CAC CCG CTA CCT GGT GTT AG-3′, Reverse 5′-GGT CAT CCG TGT GAA GCA G-3′). AMH gene (2) (Forward 5′-AAG CTG CTC ATC AGC CTG TC-3′, Reverse 5′-TGG GGT CCG AAT AAA TAT GG-3′). AMHR-II gene (1) (Forward 5′-CCC TGC TAC AGC GAA AGA AC-3′, Reverse 5′-ATG GCA ACC AGT TTT CCT TG-3′). AMHR-II gene (2) (Forward 5′-AAC TGG CCT ATG AGG CAG AA-3′, Reverse 5′-GGT CTG CAT CCC AAC AGT CT-3′). GAPDH gene (Forward 5′-GGA GTC AAC GGA TTT GGT CGT-3′, Reverse 5′-GCT TCC CGT TCT CAG CCT TGA-3′).

## Results

**Figure 1:**
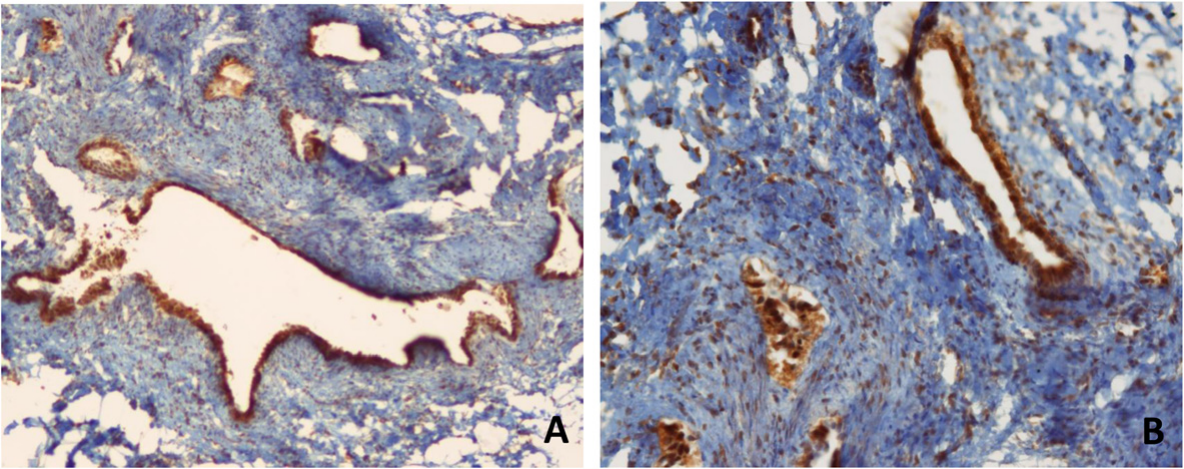
**Immunohistochemical expression of AMH in endometriosis tissues. (A)** AMH expression in the epithelium of an endometriosis gland (Original magnification X20). **(B)** The immunohistochemical expression of AMH is clearly visible also in the stromal cells of the endometriosis gland (Original magnification X20).

Histologic examination of endometriosis lesions of the rectovaginal septum showed the typical presence of both endometriotic glands and stroma. Immunohistochemical staining demonstrated that both epithelial and stromal component expressed significant levels of AMH. Figure 1 depicts some exemplary cases of the immunohistochemical staining for AMH in cases of endometriosis of the rectovaginal septum.

**Figure 2:**
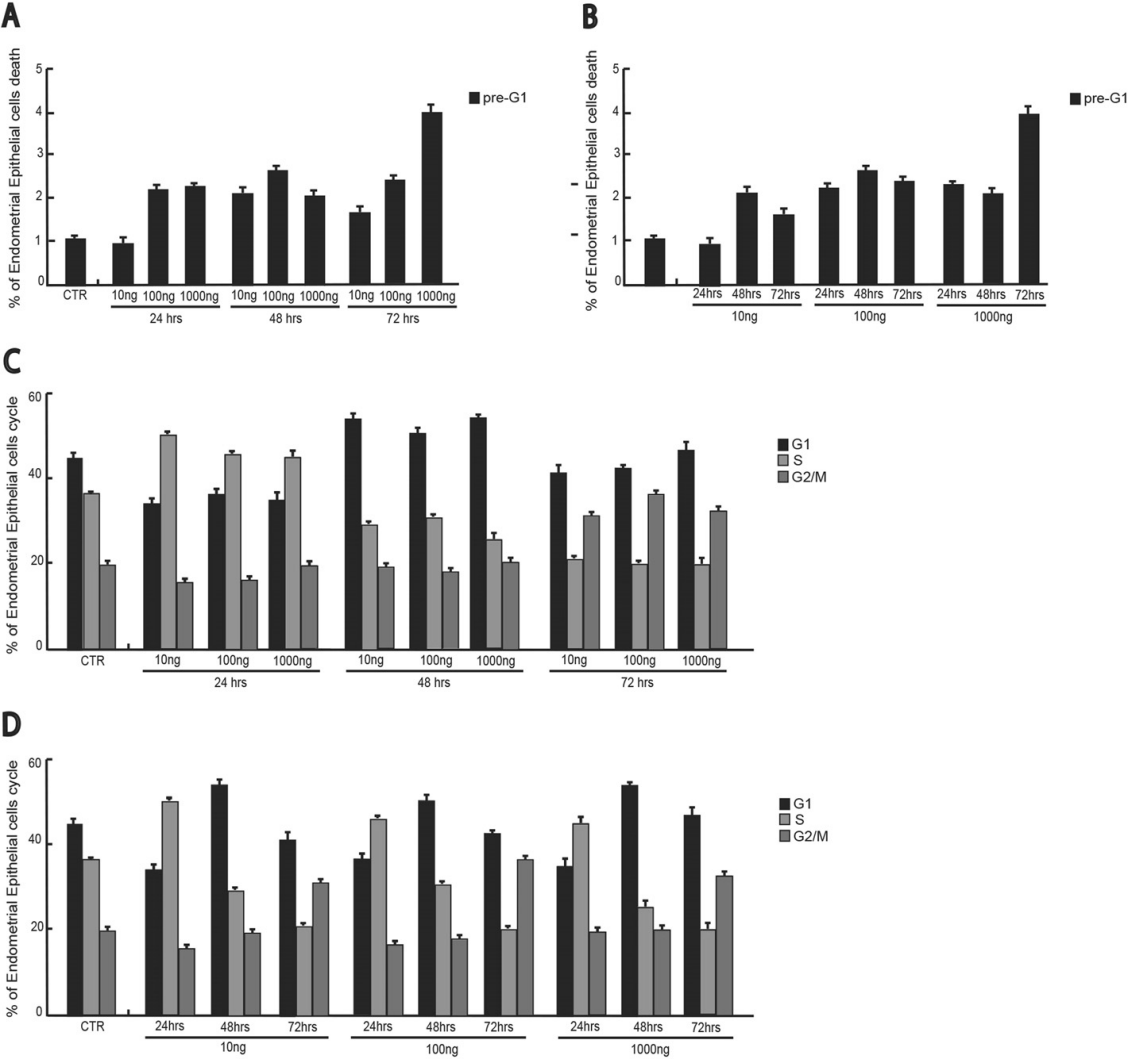
**Effects of recombinant human Mullerian-inhibiting substance (MIS)/anti-Mullerian hormone (E.Coli derived) on endometriosis epithelial cell line. (A)** pre-G1 fraction analysis of endometriosis epithelial cells treated for 24-48-72 hrs with the indicated final concentrations of MIS. The data are shown in a time-dependent manner. **(B)** pre-G1 fraction analysis of endometriosis epithelial cell line treated for 24-48-72 hrs with the indicated final concentrations of MIS. The data are shown in a dose-dependent manner. **(C)** Cell cycle analysis of endometriosis epithelial cells treated 24-48-72 hrs with the indicated final concentrations of MIS. The data are shown in a time-dependent manner. **(D)** Cell cycle analysis of endometriosis epithelial cells treated 24-48-72 hrs with the indicated final concentrations of MIS. The data are shown in a dose-dependent manner.

**Figure 3:**
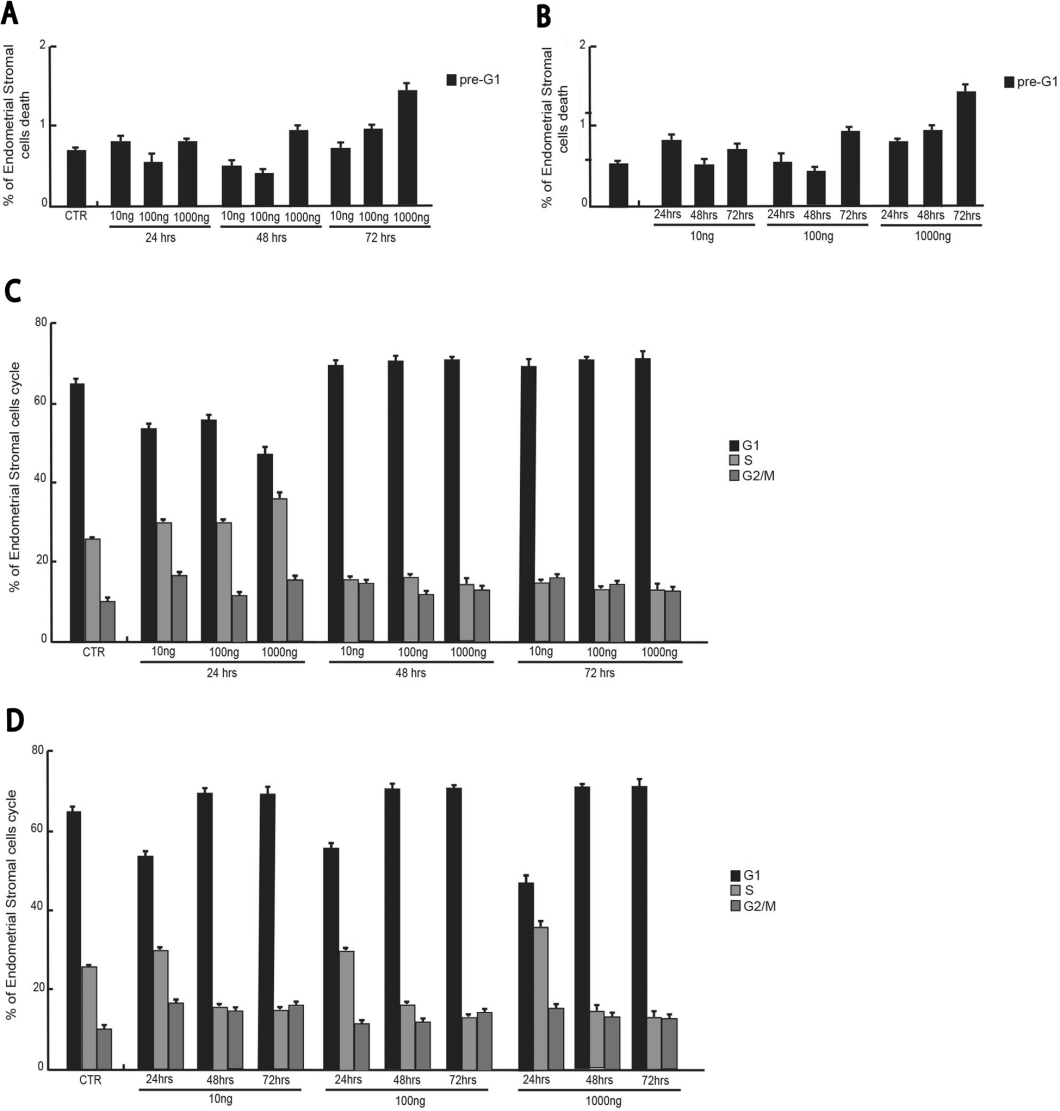
**Effects of recombinant human Mullerian-inhibiting substance (MIS)/anti-Mullerian hormone (E.Coli derived) on endometriosis stromal cell line. (A)** pre-G1 fraction analysis of endometriosis stromal cells treated for 24-48-72 hrs with the indicated final concentrations of MIS. The data are shown in a time-dependent manner. **(B)** pre-G1 fraction analysis of endometriosis stromal cell line treated for 24-48-72 hrs with the indicated final concentrations of MIS. The data are shown in a dose-dependent manner. **(C)** Cell cycle analysis of endometriosis stromal cells treated for 24-48-72 hrs with the indicated final concentrations of MIS. The data are shown in a time-dependent manner. **(D)** Cell cycle analysis of endometriosis stromal cells treated for 24-48-72 hrs with the indicated final concentrations of MIS. The data are shown in a dose-dependent manner.

**Figure 4:**
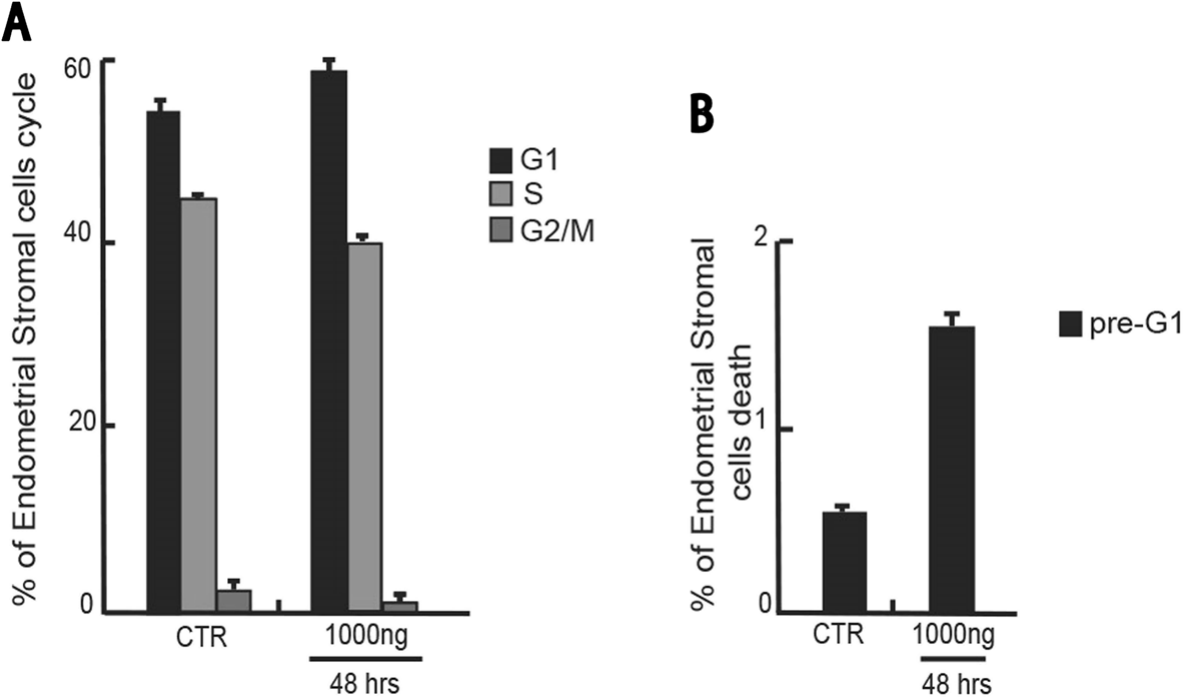
**Effects of purified recombinant protein of Homo Sapiens anti-Mullerian hormone (AMH) on endometriosis stromal cell line. (A)** Cell cycle analysis of endometriosis stromal cells treated for 48 hrs with AMH at 1000 ng/mL. **(B)** pre-G1 fraction analysis of endometriosis stromal cells treated for 48 hrs with AMH at 1000 ng/mL.

**Figure 5:**
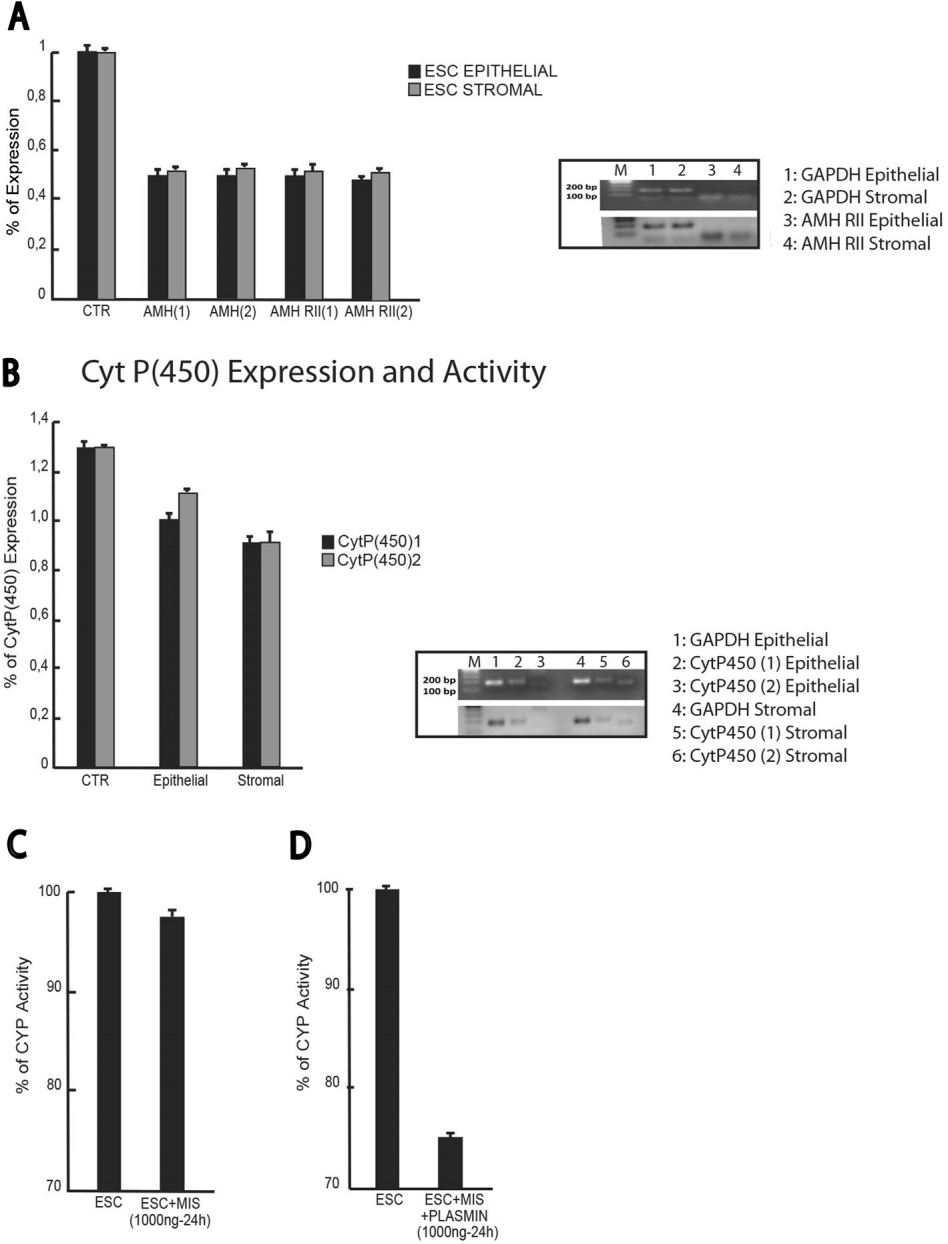
**Analysis of AMH, AMHRII expression and CytP450 activity. (A)** Real-time PCR to assess the percentage of expression levels of AMH (1), AMH (2), AMH type II Receptor (1) and (2) (AMH RII) genes in endometrial epithelial and stromal cell line respectively. **(B)** Expression levels of the Cytochrome P4501 and 2 isoforms and Reverse Transcriptase–Polymerase Chain Reaction (RT-PCR) for the CytP (450) 1 and 2 in epithelial and stromal cell line respectively; GAPDH represents loading control. **(C)** CYP Activity assay in endometrial stromal cells treated for 24 hrs at 1000 ng/mL of MIS full-length. **(D)** CYP Activity assay in endometrial stromal cells treated for 24 hrs at 1000 ng/mL of Plasmin-cleaved MIS.

We were able to demonstrate the effects induced by Recombinant Human Mullerian-Inhibiting Substance (rhMIS)/anti-Mullerian hormone (AMH)E. Coli derived on endometriosis stromal and epithelial cell growth, cell cycle progression and apoptosis induction. We have treated cultured human endometriosis stromal and epithelial cells with rhMIS at different concentrations (10-100-1000 ng/mL) and analyzed the effects induced after 24-48-72 hours of treatment.To investigate the various phases of the cell cycle, the Flow Cytometer FACS (Fluorescent Activated Cell Sorter) was used. The results shown were obtained using the Cell Quest software (Becton Dickinson) and are shown in a dose and time dependent manner to better visualize the effect induced by treatment (Figure 2). As depicted in Figure 2A and B, induction of cell death was present upon treatment in a time and dose dependent manner. Despite weak, this apoptotic effect was fully reproducible and specifically connected to the hormone treatment. The changes in cell cycle distribution after 24 hours of AMH exposure suggested that AMH plays an important role in inducing an initial increase in the percentage of cells in the S phase, which is translated into a G1 block at 48 hrs. Interestingly, while the effects on apoptosis are dose and time dependent, the cell cycle effects seem only time dependent (Figure 2C-D). The results of high-AMH concentrations treatment have confirmed a decreased percentage of cells in S phase with increased percentage of cells in G1 and G2 phase (Figure 2D) and increasing local AMH concentration in cultured human endometriosis stromal cells decreased cell viability and increased percentage of cells death fraction also (Figure 2A-B).These effects where fully confirmed by using the stromal cells (Figure 3). Despite slightly more resistant, in these cells the apoptosis induced by the hormone was time and dose dependent, whereas the cell cycle effects were only time dependent.Similarly, the Purified recombinant protein of Homo sapiens AMH treatment (10-100-1000 ng for 24-48-72 hours) on endometriosis stromal cells line resulted in coherent results (Figure 4A-B). A small decrease in percentage of cells in S and G2/M phases was observed (Figure 4A) with a concomitant increase of cells in pre-G1 phase (Figure 4 B).Various semi-quantitative RT-PCR have been used to quantify the expression levels of AMH and AMH RII isoforms in both endometriosis epithelial and stromal cells (Figure 5A). The two isoforms analyzed were designed with the Primer3 software. Both endometriosis epithelial and stromal cells expressed mRNA for AMH and AMH RII (Figure 5A). Finally, the expression levels of CYP19 were confirmed through real-time PCR analysis (Figure 5B).

**Figure 6:**
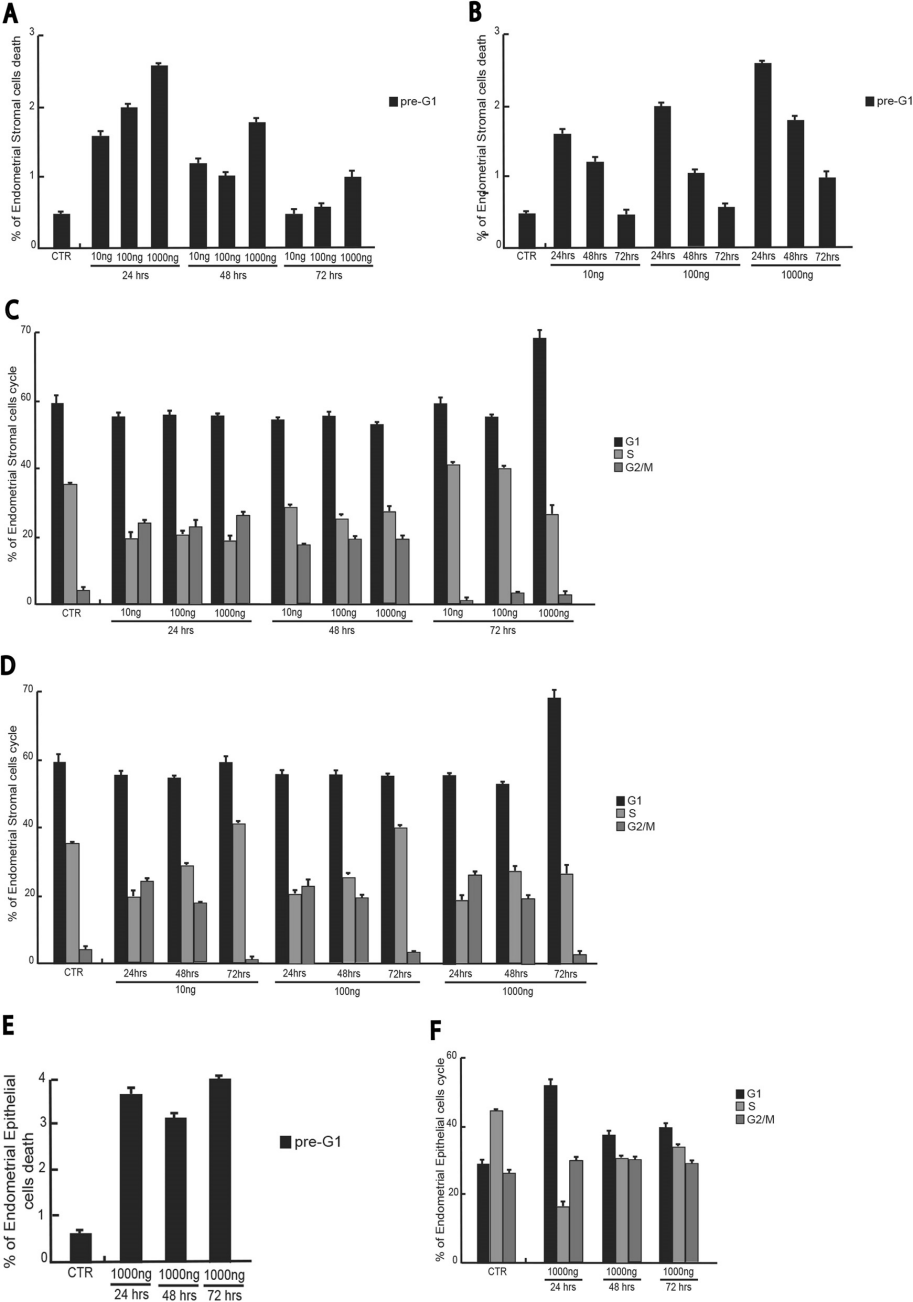
**Effects of purified recombinant protein of Homo Sapiens anti-Mullerian hormone (AMH) digested by Plasmin from human plasma on endometriosis stromal and epithelial cell line. (A)** pre-G1 fraction analysis of endometriosis stromal cells treated for 24-48-72 hrs with the final concentrations of cleaved MIS as indicated. The data are shown in a time-dependent manner. **(B)** pre-G1 fraction analysis of endometriosis stromal cells treated for 24-48-72 hrs with the final concentrations of cleaved MIS as indicated. The data are shown in a dose-dependent manner. **(C)** Cell cycle analysis of endometriosis stromal cells treated for 24-48-72 hrs with the final concentrations of cleaved MIS as indicated. The data are shown in a time-dependent manner. **(D)** Cell cycle analysis of endometriosis stromal cells treated for 24-48-72 hrs with the final concentrations of cleaved MIS as indicated. The data are shown in a dose-dependent manner. **(E)** pre-G1 fraction analysis of endometriosis epithelial cells treated for 24-48-72 hrs with 1000 ng/mLof cleaved MIS. The data are shown in a time-dependent manner. **(F)** Cell cycle analysis of endometriosis epithelial cells treated for 24-48-72 hrs with 1000 ng/mLof cleaved MIS. The data are shown in a time-dependent manner.

Considering that the plasmin-digested AMH has been reported to be more active in cultured human endometrial cell lines [15], human plasmin was used to cleave and activate the recombinant Human AMH at its monobasic arginine-serine site at residues 427-428 and then tested in functional experiments on both endometriosis stromal and epithelial cells. Firstly, we found that plasmin-digested AMH can alter the expression or function of CYP19, evaluated by testing CYP19 activity. The results suggest that the plasmin-digested AMH was able to suppress most of the CYP19 activity. When the plasmin-digested AMH was used on both endometriosis stromal and epithelial cells (Figure 6), an increase of pre-G1 phase treating with plasmin-digested AMH in both cell lines was detected, most marked in the epithelial cells (Figure 6). Also the effect on induction of apoptosis was stronger during the first 24 hours of treatment (Figure 6A-B). Interestingly, and differently from the previous experiments, apoptosis decreased after the 24 hrs, suggesting that, possibly, the cleaved AMH is more unstable than the full-length protein. These results are also supported by the cell cycle analysis which showed very weak effects on the stromal cells, only at 72 hrs at the highest concentration (Figure 6E-F).

## Discussion

Endometriosis is a benign disease of women during reproductive age [17]; nevertheless, it is well known that endometriosic cells display functional properties that are typical of neoplastic cells, such as anti-apoptotic, invasive and metastatic capacities [18,19]. In support to this observation, epidemiological studies have shown that there exists an increased risk of different types of malignancies, especially ovarian cancer and non-Hodgkin’s lymphoma in women with endometriosis [20,21]. Nevertheless, it has been reported an association between endometriosis, dysplastic nevi, melanoma, and breast cancer [22,23]. Finally, several histological and genetic studies have indicated that endometriosis may transform into cancer or that it could be considered a precursor of cancer [24]. Recently, it has been demonstrated by Wang et al., that adult human endometrium has a functional AMH/AMHRII signal transduction system and that the activation of this system is able to negatively regulate cellular viability in cultured endometrial cells [12]. Indeed, the exact biological role of AMH in adult females is unclear. The most well recognized function in adult is its involvement in recruitment and selection of initial primordial follicles [25]. In fact, there exists a plethora of research articles on the use of AMH serum level as a sensitive marker to assess the ovarian reserve [26]. However, consistently with the work by Wang et al. [12], several papers have shown a part for AMH as a regulator of cell growth in cells and tissues of Mullerian origins, such as endometrial, ovarian, cervical and breast tissues and a role for AMH as potential therapeutic factor in tumors originating from these tissues has been proposed [27-31]. Recently, two independent research groups have demonstrated that the AMH system is active also in endometriosic cells in vitro and that it acts as a negative regulator of cell cycle and cell viability [32,33].

In this study we have shown that AMH protein is clearly expressed in endometriosis glands in humans; that it is also expressed together with its receptor AMH RII in our in vitro model of endometriosis; and that it is able to inhibit cell proliferation and to induce apoptosis in endometriosis cells, both epithelial and stromal. Several experimental studies have revealed that AMH is strongly activated by cleavage [34]. In fact, the C-terminal fragment contains the conserved TGFβ domain [35] and the cleavage is necessary for efficient receptor binding [36]. Consistent with these observation, it has been reported that the plasmin-digested AMH is more active in cultured human endometrial cell lines [15]. In our experimental setting, we have been able to demonstrate that cleaved AMH is effective in inhibiting cell proliferation in endometriosis cells. Moreover, this cleaved form of AMH is able to inhibit most of the CYP19 activity in endometriosis cells, as it has been already shown for cultured granulosa lutein cells [15]. Several studies have suggested that endometriosis implants are able to produce estrogen *de novo* from cholesterol [37]. Therefore, endogenous steroidogenic genes in local estradiol biosynthesis in endometriosis are crucial for the survival of these implants. Based on this rationale, it has been recently proposed the use of aromatase inhibitors as a novel treatment of endometriosis. Our experimental data demonstrate, indeed, that AMH treatment is able to inhibit CYP19 activity, that is the key enzyme in humans for the conversion of C_19_ steroids to estrogens [38], thus suggesting a possible biological explanation of the effects of this hormone on cell growth and apoptosis.

## Conclusions

The clinical and therapeutic implications of this observation are straightforward. In fact, all current endometriosis treatments, including surgical and medical strategies, have high recurrence rates of up to 45% [17]. The data produced suggest a possible use of AMH as therapeutic agents in endometriosis. Additional functional studies both in vitro and in vivo are necessary in order to define applicable therapeutic modalities.

## Competing interests

The authors declare that they have no competing interests.

## Authors’ contributions

PGS and AB conducted the work, analyzed the data and wrote together the manuscript; FP performed the in vitro experiments. All authors read and approved the final manuscript.
